# Bassini’s Radical Cure of Hernia

**Published:** 1892-01

**Authors:** 


### BASSINI'S RADICAL CURE OF HERNIA.

At the recent congress of German surgeons, Dr. Escher, of Trieste, reported his results from this method of operation. It consists in laying open the inguinal canal over its entire extent and somewhat beyond; the spermatic cord and hernial sac is then lifted up, and the latter incised up to its neck, and then ligated, excised and the remaining portion replaced. The layers of the internal oblique and transversalis muscles are then carefully isolated, as well as the internal portion of Poupart's ligament, and the edges of these structures are accurately united by suture. The spermatic cord is saturated in this gutter, and the fascia, muscles and skin united. The author has employed this method in 53 herniæ. The results were as follows: Of the 35 cases of reducible herniae, 25 per cent. healed by first intention, 10 with suppuration; of the nine cases of incarcerated hernia, 5 healed by first intention, 3 with suppuration, and one terminated fatally; of the 9 cases of irreducible hernia, 4 healed by primary union, 4 by suppuration and one died. As regards the permanence of the cure, in 24 cases which can be utilized for this purpose, there have been no recurrences during a period of observation varying from three months to two years.—*Deutsche Medicinische Wochenschr.*—*Toledo Med. Compend.*

